# *QuickStats*: Age-Adjusted Homicide Rates,*^,†^ by Race/Ethnicity — National Vital Statistics System, United States, 2015–2016

**DOI:** 10.15585/mmwr.mm6715a8

**Published:** 2018-04-20

**Figure:**
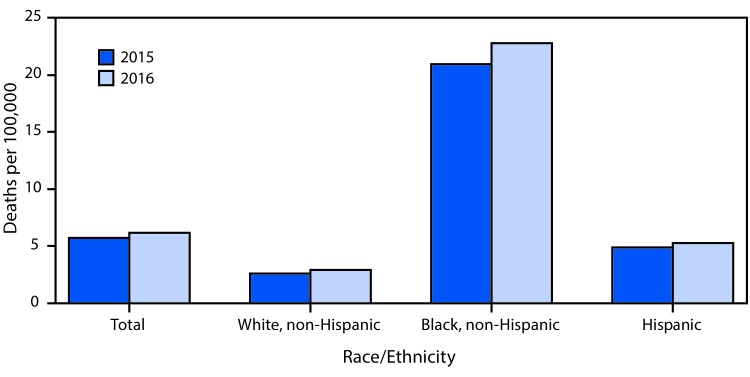
During 2015–2016, the age-adjusted homicide rate for the total population increased from 5.7 to 6.2 per 100,000 standard population (an 8.8% increase). The rate increased from 2.6 to 2.9 (11.5%) for non-Hispanic whites, from 20.9 to 22.8 (9.1%) for non-Hispanic blacks, and from 4.9 to 5.3 (8.2%) for Hispanics. In both years, the homicide rate for non-Hispanic blacks was approximately eight times the rate for non-Hispanic whites and four times the rate for Hispanics.

**Source:** National Vital Statistics System, underlying cause of death data, 1999–2016. https://wonder.cdc.gov/ucd-icd10.html.

For more information on this topic, CDC recommends the following link: https://www.cdc.gov/violenceprevention/index.html.

